# Mycobacterial Caseinolytic Protease Gene Regulator ClgR Is a Substrate of Caseinolytic Protease

**DOI:** 10.1128/mSphere.00338-16

**Published:** 2017-03-15

**Authors:** Yoshiyuki Yamada, Thomas Dick

**Affiliations:** a Department of Medicine, Yong Loo Lin School of Medicine, National University of Singapore, Singapore; b Department of Microbiology and Immunology, Yong Loo Lin School of Medicine, National University of Singapore, Singapore; Washington University in St. Louis School of Medicine

**Keywords:** ClgR, *Mycobacterium tuberculosis*, antimicrobial agents, bortezomib, caseinolytic protease, mechanisms of action

## Abstract

The mycobacterial caseinolytic protease ClpP1P2 is a degradative protease that recently gained interest as a genetically and pharmacologically validated drug target for tuberculosis. The first whole-cell active ClpP1P2 inhibitor, the human proteasome inhibitor bortezomib, is currently undergoing lead optimization to introduce selectivity for the bacterial target. How inhibition of ClpP1P2 translates into whole-cell antimicrobial activity is little understood. Previous work has shown that the caseinolytic protease gene regulator ClgR is an activator of the *clpP1P2* genes and also suggested that this transcription factor may be a substrate of the protease. Here, we employ promoter activity reporters and direct mRNA level measurements showing that bortezomib treatment of *Mycobacterium bovis* BCG increased transcription of *clpP1P2* and other ClgR-dependent promoters, suggesting that inhibition of ClpP1P2 increases cellular ClgR levels. Then, we carried out red fluorescent protein-ClgR fusion analyses to show that ClgR is indeed a substrate of ClpP1P2 and to identify ClgR’s C-terminal nonapeptide APVVSLAVA as the signal sufficient for recognition and efficient protein degradation by ClpP1P2. Interestingly, accumulation of ClgR appears to be toxic for bacilli, suggesting a mechanism for how pharmacological inhibition of ClpP1P2 protease activity by bortezomib translates into whole-cell antibacterial activity.

**IMPORTANCE** With 9 million new cases and more than 1 million deaths per year, tuberculosis, caused by *Mycobacterium tuberculosis*, is the biggest infectious disease killer globally. New drugs for the treatment of the drug-resistant forms of the disease are needed. Recently, a new target-lead couple, the mycobacterial protease ClpP1P2 and the human anticancer drug bortezomib, was identified. However, we know little about how expression of this protease is regulated, which proteins in the bacterium it degrades, how the protease recognizes its target proteins, and how the inhibition of ClpP1P2 exerts whole-cell antimicrobial activity. Here, we show that the ClpP1P2 protease regulates its own expression, and we identified a new substrate and a new substrate recognition sequence and a mechanism for how ClpP1P2 inhibition causes bacterial growth inhibition.

## Abstract (precis)

With 9 million new cases and more than 1 million deaths per year, tuberculosis, caused by *Mycobacterium tuberculosis*, is the biggest infectious disease killer globally. New drugs for the treatment of the drug-resistant forms of the disease are needed. Recently, a new target-lead couple, the mycobacterial protease ClpP1P2 and the human anticancer drug bortezomib, was identified. However, we know little about how expression of this protease is regulated, which proteins in the bacterium it degrades, how the protease recognizes its target proteins, and how the inhibition of ClpP1P2 exerts whole-cell antimicrobial activity. Here, we show that the ClpP1P2 protease regulates its own expression, and we identified a new substrate and a new substrate recognition sequence and a mechanism for how ClpP1P2 inhibition causes bacterial growth inhibition.

## OBSERVATION

The mycobacterial caseinolytic protease (Clp) is, similarly to the human proteasome, a degradative protease machine with a role in proteome housekeeping (1, 2). One of the functions of Clp is the removal of aborted translation products which have been cotranslationally tagged with the 11-amino-acid SsrA recognition sequence (3). Recently, the first endogenous substrates of the protease, the transcription factors WhiB1 and CarD, were identified, and a role for the protease in posttranslational regulation was established (2). The Clp protease complex is composed of a degradative chamber made of two different serine protease subunits, ClpP1 and ClpP2, encoded by the *clpP1P2* operon, which interacts with unfoldases involved in recognition and delivery of proteins into the degradation chamber (4).

ClpP1P2 and unfoldases are genetically *in vitro-* and *in vivo*-validated targets in *Mycobacterium tuberculosis* (1, 5–8). Recently, we developed and employed a novel screening concept, a target mechanism-based whole-cell assay, to identify the first whole-cell active inhibitor of the mycobacterial ClpP1P2, bortezomib (9). Bortezomib, an anticancer drug, inhibits the human proteasome via binding to its protease catalytic sites (10). We showed via ClpP1P2 under- and overexpression studies, 50% inhibitory concentration (IC_50_)-MIC_50_ structure-activity relationship correlation studies, and structural analyses that it is indeed on the target, i.e., via inhibition of the ClpP1P2 protease (and not other cellular targets), that bortezomib exerts its antibacterial whole-cell activity (9). The exact mechanisms, the intracellular follow-on events (11), and how pharmacological inhibition of ClpP1P2 translates into growth inhibition remain to be established.

Recently, Sherman and colleagues as well as Stewart and colleagues showed that *clpP1P2* expression in *M. tuberculosis* is under positive control by the transcriptional activator ClgR (12, 13). The orthologue of mycobacterial ClgR had previously been identified in *Streptomyces lividans*, where it was named “caseinolytic protease gene regulator,” ClgR (14). Furthermore, Mazodier and colleagues showed that missense mutations in the last two C-terminal amino acids (AA to DD) stabilized the ClgR protein and enhanced *clpP1P2* expression, indicating a possible role of the C terminus of ClgR in recognition by *Streptomyces* Clp (15). Mutating the same two amino acid positions (VA to DD) in *M. tuberculosis* ClgR also stabilized the ClgR protein and enhanced *clpP1P2* expression, suggesting a similar mechanism in mycobacteria (12). Taken together, these works suggested that the *clpP1P2* activator ClgR may be a substrate of ClpP1P2 in mycobacteria.

**FIG 1:**
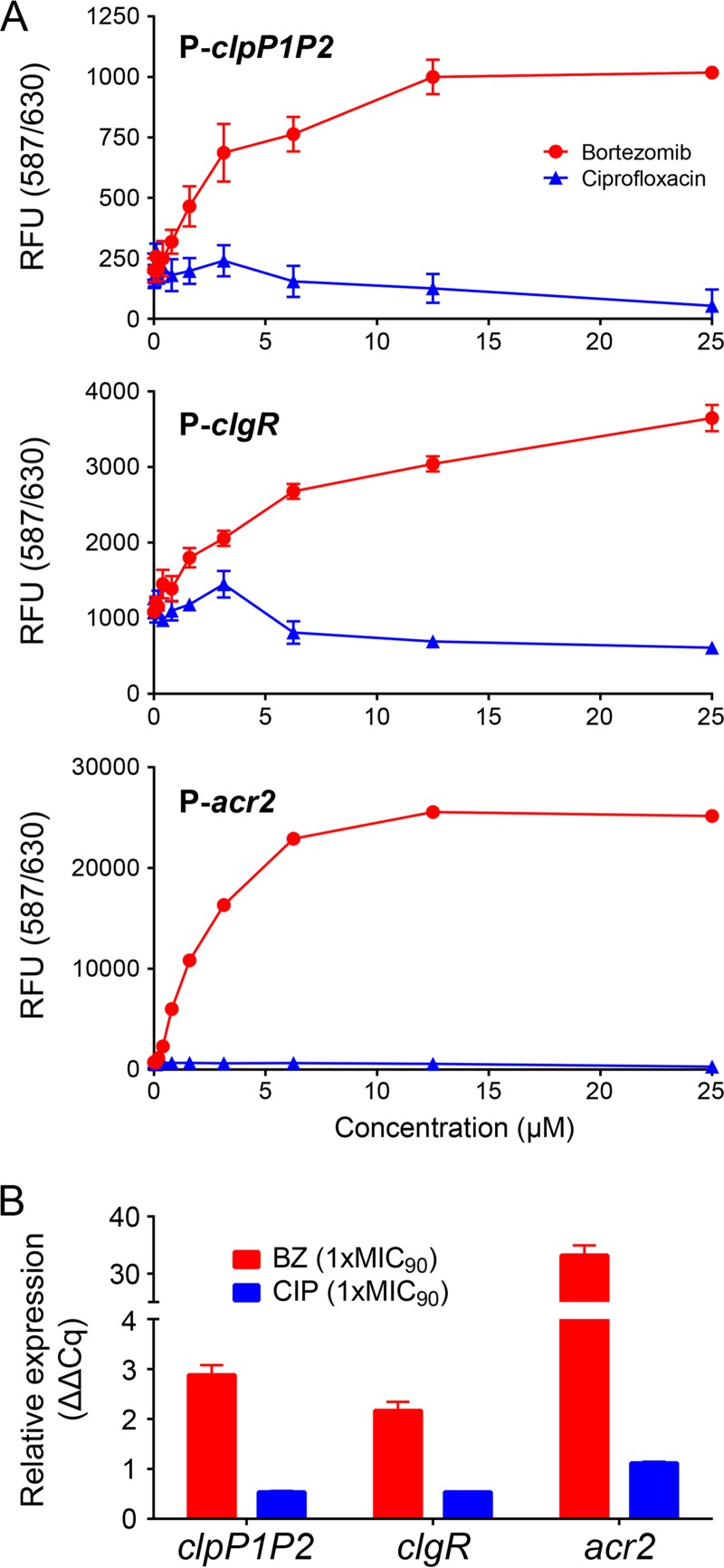
Bortezomib treatment increases transcription of *clpP1P2*, *clgR*, and *acr2* genes in *M. bovis* BCG. (A) Bortezomib dose-dependent increase of RFP expression under the control of P-*clpP1P2*, P-*clgR*, and P-*acr2* promoters after 24 h of bortezomib treatment. RFU, relative fluorescence units. Primers and plasmid construction procedures using the integrative plasmid pMV306 (18) to generate the respective reporter strains are listed in Table S1 in the supplemental material. OD_600_ was measured during the course of the experiment and was found to increase a maximum of 2-fold in the drug-free samples and less in the drug-containing samples. (B) Bortezomib-dependent increase of *clpP1P2*, *clgR*, and *acr2* mRNA. Transcript levels were measured after 16 h of bortezomib treatment. Primer sequences (16, 19) can be found in Table S2 in the supplemental material. Relative expression (quantification cycle [ΔΔ*C*_*q*_]) was calculated as described previously (20) by using 16S RNA as the reference. BZ, bortezomib. CIP, ciprofloxacin. MIC_90_, drug concentration that inhibited growth of the bacteria by 90%. MIC_90_ of BZ, 12.5 µM. MIC_90_ of CIP, 1.6 µM. Data in panels A and B are represented as means ± standard deviations from two biological and four technical replicates.

Here, we wanted to determine whether mycobacterial ClgR is indeed a Clp substrate and, if yes, identify the sequence that targets ClgR for degradation. In addition to being a transcriptional activator of *clpP1P2*, ClgR positively regulates several other genes, including its own gene, *clgR*, as well as *acr2*, encoding a chaperon (12, 13, 16). Thus, if ClgR is a substrate of Clp, pharmacological inhibition of the protease by bortezomib should result in an increase of ClgR levels and therefore increased transcription of ClgR-dependent promoters. Figure 1 shows that this is the case. Bortezomib treatment increased activity of the P-*clpP1P2*, P-*clgR*, and P-*acr2* promoters as visualized by a dose-dependent increase of red fluorescent protein (RFP; mCherry) expression in the respective *Mycobacterium bovis* BCG reporter strains (Fig. 1A). Bortezomib treatment also increased the levels of the mRNA for *clpP1P2*, *clgR*, and *acr2* in *M. bovis* BCG wild-type cultures (Fig. 1B). These effects were bortezomib specific, as treatment of the cultures with the gyrase inhibitor ciprofloxacin did not result in increased promoter activities or elevated transcript levels (Fig. 1A and B). Taken together, bortezomib-dependent coactivation of the ClgR-dependent promoters suggested that bortezomib treatment increases ClgR levels and, by implication, that ClgR may be a substrate for Clp.

**FIG 2:**
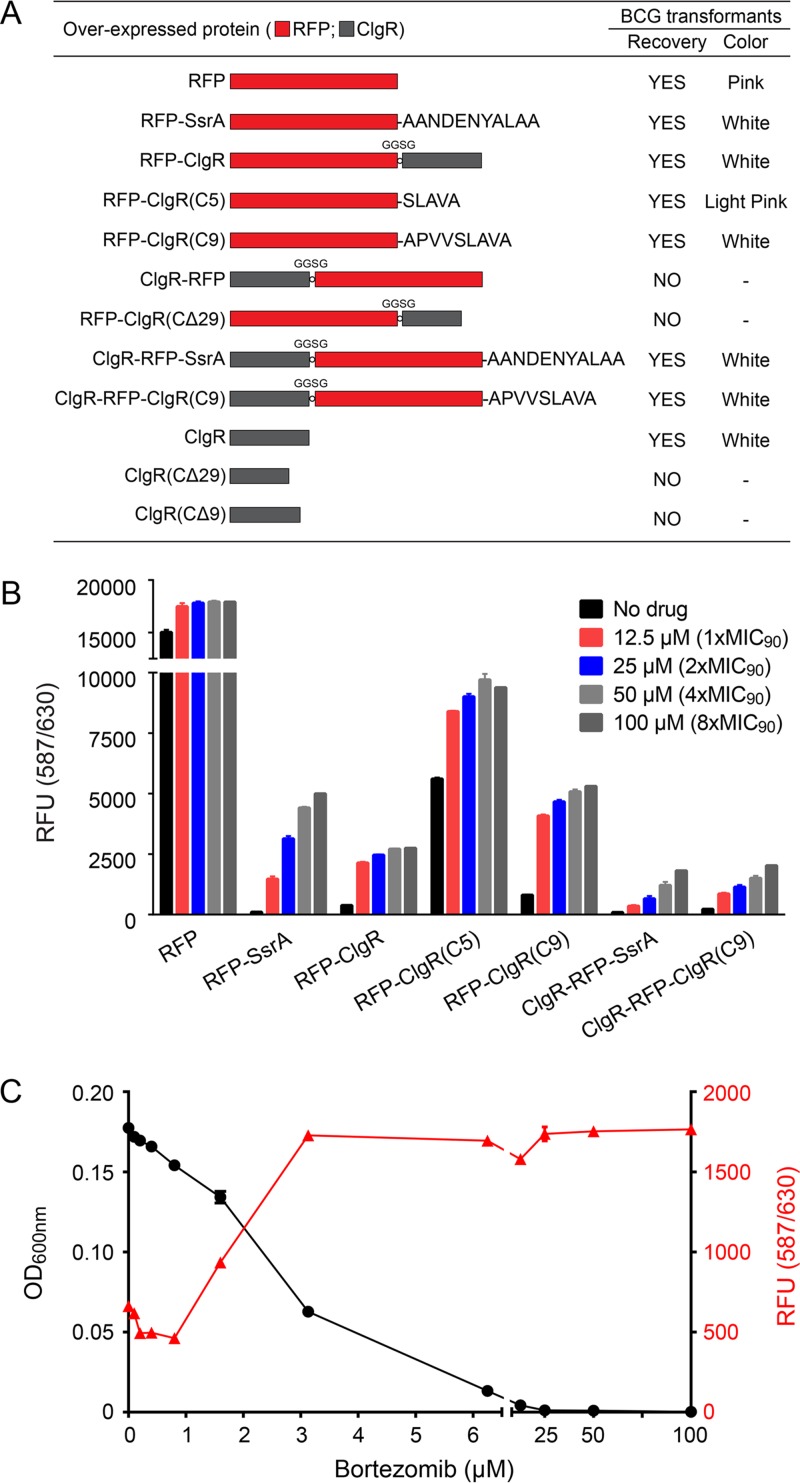
ClgR is a substrate of mycobacterial Clp, and its accumulation is toxic for *M. bovis* BCG. (A) Schematics of ClgR-RFP fusion proteins, their transformability, and colony color of *M. bovis* BCG transformants. Red, RFP; gray, ClgR. “Recovery” indicates whether transformants with the respective constructs could be obtained. “Color” indicates the color of the *M. bovis* BCG colonies. “GGSG” indicates the peptide sequence used as a short linker inserted between RFP and ClgR. Primers used and plasmid construction procedures employing the episomal plasmid pMV262 (18) to generate the respective strains are listed in Table S3 in the supplemental material. All ClgR-RFP fusion proteins as well as the ClgR nonfusion proteins were overexpressed in *M. bovis* BCG under the control of the constitutive P-*hsp60* promoter (17). Note that the transformation efficiencies for the overexpression constructs for which colonies could be recovered were in the range of 5 × 10^4^/µg DNA and were similar for all constructs. Colony sizes were also similar with the exception of RFP-ClgR, ClgR-RFP-SsrA, and ClgR-RFP-ClgR(C9) transformants, which displayed somewhat smaller colony sizes. For overexpression constructs for which no transformants were obtained, the plates were incubated and observed for 2 months. (B) Fluorescence measurements of *M. bovis* BCG cultures carrying various ClgR-RFP fusion constructs shown in panel A without and with 24-h bortezomib treatment. (C) Effect of increasing bortezomib concentrations on fluorescence and growth of *M. bovis* BCG cultures expressing the RFP–full-length ClgR fusion protein (RFP-ClgR [A]). RFU, relative fluorescence units. The bacteria were grown in 96-well plates for 5 days as described in the text with a starting OD_600_ of 0.05. Turbidity and fluorescence measurements were taken after day 5 with an Infinite M200 Pro plate reader (Tecan). Data shown in panels B and C represent means ± standard deviations from two biological and four technical replicates.

To date, the *trans*-translation SsrA tag (1) and the transcription factors WhiB1 and CarD (2) have been characterized in detail as the substrates of Clp in mycobacteria. In all cases, a C-terminal 5- to 15-amino-acid sequence has been implicated as the recognition signal required and sufficient for degradation by the protease machinery. To determine whether ClgR is indeed a substrate of ClpP1P2, we therefore fused the ClgR protein to the C terminus of RFP. Figure 2A shows that *M. bovis* BCG expressing unmodified RFP showed pink colonies and a high level of fluorescence when grown in broth culture, reflecting high levels of intracellular RFP (Fig. 2B). In contrast, RFP tagged with the Clp degradation tag SsrA was effectively degraded in *M. bovis* BCG (RFP-SsrA) and showed white colonies with minimal signals of RFP fluorescence (Fig. 2A and B). If ClgR is recognized and degraded by ClpP1P2, fusion of this protein to RFP should result in degradation of the RFP-ClgR fusion protein and hence a loss of RFP fluorescence. Figure 2A shows that the respective *M. bovis* BCG RFP-ClgR strain indeed grew white colonies and that the corresponding broth cultures showed only a background level of fluorescence (Fig. 2B), suggesting that RFP-ClgR is degraded. If degradation of RFP-ClgR is ClpP1P2 dependent, bortezomib treatment should result in an increase of fluorescence. Figure 2B shows that bortezomib treatment of RFP-ClgR cultures indeed resulted in an increase of fluorescence. Taken together, these results suggest that ClgR is recognized as the substrate of ClpP1P2 and degraded by this protease.

The recognition sequences of currently known Clp substrates are short C-terminal peptides: AANDENYALAA for SsrA-tagged proteins, ARTGV for WhiB1, and AKAETILDEVLAAAS for CarD (2). To determine whether the ClgR degradation signal is also provided by its C terminus, we fused the last 5 [SLAVA; RFP-ClgR(C5)] and 9 [APVVSLAVA; RFP-ClgR(C9)] amino acids of ClgR to the C terminus of RFP. Figure 2A shows that the *M. bovis* BCG RFP-ClgR(C5) colonies were slightly pink, whereas *M. bovis* BCG RFP-ClgR(C9) colonies were white. Correspondingly, *M. bovis* RFP-ClgR(C5) broth cultures showed high levels of fluorescence, an indication of poor degradation of the RFP carrying only the last 5 amino acids of ClgR. In contrast, cultures of *M. bovis* RFP-ClgR(C9) showed a background level of fluorescence, similarly to cultures of *M. bovis* BCG expressing the RFP–full-length ClgR fusion, indicating efficient degradation of RFP-ClgR(C9) fusion protein (Fig. 2B). Taken together, these results suggest that the C-terminal ClgR pentapeptide SLAVA is only weakly recognized as a substrate, whereas the nonapeptide APVVSLAVA is an effective degradation signal for the mycobacterial Clp.

Interestingly, attempts to construct a strain overexpressing ClgR as an N-terminal fusion of RFP (in which ClgR cannot be recognized as the substrate by Clp) were not successful, i.e., *M. bovis* BCG colonies could not be recovered when transformed with the corresponding plasmid constructs (Fig. 2A). Similar results were obtained when we overexpressed enhanced green fluorescent protein (eGFP)-ClgR or ClgR-eGFP in *M. bovis* BCG. eGFP-ClgR fusion-expressing strains could be obtained, whereas N-terminal ClgR-eGFP fusions appeared to be toxic and transformants could not be generated (see Table S3 in the supplemental material; also data not shown). Attempts to construct C-terminal fusions of ClgR to RFP with the C terminus of ClgR, including the nonapeptide recognition sequence, deleted but with an intact helix-turn-helix DNA binding domain of the transcription factor (ClgRΔC29 [Fig. 2A]) also did not yield viable *M. bovis* BCG transformants. These results suggest that increased levels of a functional transcription factor, ClgR, may be toxic to the bacteria. If indeed the increased levels of ClgR-RFP are toxic and therefore prevent generation of these strains, attaching a degradation tag to the C terminus of ClgR-RFP should allow recovery of viable bacteria and therefore construction of the corresponding strain. Figure 2A shows that this is the case. Attaching the SsrA degradation tag (ClgR-RFP-SsrA) or the newly identified ClgR degradation tag [ClgR-RFP-ClgR(C9)] to the C terminus of ClgR-RFP allowed recovery of (white) colonies (Fig. 2A), and respective broth cultures showed background-level fluorescence—which was increased by bortezomib treatment (Fig. 2B).

To provide fluorophore-fusion-independent evidence that increased levels of ClgR are toxic for the bacteria and could be a possible mechanism by which bortezomib exerts its antibacterial effect, we attempted to express non-fluorophore-fusion versions of ClgR with and without the Clp recognition sequence. Figure 2A shows that an *M. bovis* BCG strain overexpressing the full-length ClgR protein (containing the Clp recognition sequence) could be constructed. In contrast, when we transformed plasmids overexpressing C-terminal truncations of ClgR (lacking the Clp recognition sequence), we were unable to obtain transformants (Fig. 2A). These results mirror exactly the results from the overexpression experiments of the corresponding RFP-ClgR fusion proteins (Fig. 2A) and suggest that toxicity is due to the accumulation of nondegradable ClgR protein (and not due to the toxicity of protein-fluorophore fusions).

If increasing the level of ClgR is indeed toxic to *M. bovis* BCG, we expect an inverse relationship between growth and ClgR level. To test this hypothesis, we subjected *M. bovis* BCG carrying ClgR fused to the C terminus of RFP (RFP-ClgR [Fig. 2A]) to increasing concentrations of bortezomib and measured growth of the culture and fluorescence, i.e., RFP-ClgR level. Figure 2C shows an inverse relationship between growth and RFP-ClgR levels. A bortezomib concentration (2 µM) causing around half-maximum ClgR level increase (measured as increase of fluorescence of RFP-ClgR) caused about half-maximum growth inhibition. These results suggest that accumulation of ClgR is part of the mechanism of how inhibition of ClpP1P2 by bortezomib exerts whole-cell antimicrobial activity: toxic accumulation of a transcription factor. A similar observation was made for the transcription factor WhiB1 using genetic analyses (2).

In conclusion, we first provide evidence that the mycobacterial ClgR is a substrate of ClpP1P2. Thus, we identified a novel regulatory feedback loop in mycobacteria: ClpP1P2 controls its own expression by regulating the level of the transcriptional activator of its encoding genes. Second, we identified the C-terminal nonapeptide APVVSLAVA of ClgR as a novel degradation tag recognized by Clp. Finally, we show that accumulation of ClgR appears to be toxic for the bacteria, and thus we provide a mechanism for how inhibition of ClpP1P2 by the new lead compound bortezomib (http://www.newtbdrugs.org/pipeline/discovery) may cause inhibition of growth of the organism.

## 

### Strains and culture conditions.

*M. bovis* BCG Pasteur ATCC 35734 (BCG) was purchased from the American Type Culture Collection and was grown at 37°C in Middlebrook 7H9 broth (BD Difco) supplemented with 0.5% albumin, 0.2% glucose, 0.085% sodium chloride, 0.0003% catalase, 0.2% glycerol, and 0.05% Tween 80. Genomic DNA was isolated from *M. bovis* BCG as described previously (17). Primers used for plasmid constructions and respective manipulation procedures are summarized in Tables S1 and S3. PCR amplification was performed with KOD FX Neo DNA polymerase (Toyobo) according to the manufacturer’s instructions. For generating electrocompetent cells, BCG was grown at 37°C in 7H9 until the optical density at 600 nm (OD_600_) reached 0.2, 0.1 volume of 2 M glycine was added, and cells were further incubated for 16 h. Cells were washed 3 times with wash buffer (10% [vol/vol] glycerol and 0.05% Tween 80 in Milli-Q H_2_O) and resuspended in 0.02 volume of the initial culture. Electrocompetent BCG cells were mixed with 100 ng of plasmid, and electroporation was performed with a Gene Pulser apparatus (Bio-Rad) at 2,500 V, a capacity of 25 µF, and a resistance of 1,000 Ω. Bacteria were cultured overnight in fresh 7H9 and plated on 7H11 agar (BD Difco) with 25 µg/ml of kanamycin.

10.1128/mSphere.00338-16.1 TABLE S1  Reporter plasmids for measuring promoter activities and primers used in this study. Download TABLE S1, PDF file, 0.1 MB. Copyright © 2017 Yamada and Dick. 2017 Yamada and Dick This content is distributed under the terms of the Creative Commons Attribution 4.0 International license.

10.1128/mSphere.00338-16.2 TABLE S2  Primers used for qRT-PCR. Download TABLE S2, PDF file, 0.1 MB. Copyright © 2017 Yamada and Dick. 2017 Yamada and Dick This content is distributed under the terms of the Creative Commons Attribution 4.0 International license.

10.1128/mSphere.00338-16.3 TABLE S3  Plasmids to overexpress recombinant proteins and primers used in this study. Download TABLE S3, PDF file, 0.1 MB. Copyright © 2017 Yamada and Dick. 2017 Yamada and Dick This content is distributed under the terms of the Creative Commons Attribution 4.0 International license.

### Fluorescence reporter assay.

Reporter assays were carried out with dual readout, absorbance (OD_600_), and relative fluorescence units (RFU; excitation/emission [Ex/Em], 587/630 for RFP and 485/515 for eGFP, respectively) by using an Infinite M200 Pro plate reader (Tecan). Briefly, log-phase (OD_60_ of 0.4 to 0.6) *M. bovis* BCG cultures were adjusted to an OD_600_ of 0.4 in fresh 7H9, and 100 µl of cell suspension was inoculated into 96-well microplates which contained an equal volume (100 µl) of fresh 7H9 with or without drugs. After the measurement at day 0, microplates were sealed with Breathe-Easy membrane (Sigma-Aldrich) and incubated at 37°C with shaking at 80 rpm for 24 h.

### Quantitative PCR.

RNA from *M. bovis* BCG wild type was isolated from the equivalent of 20 ml of cells at an OD_600_ of 0.4. Cultures were centrifuged, resuspended in TRIzol (Invitrogen), and subjected to bead beating by using a FastPrep-24 5G instrument (MP Biomedicals; twice for 45 s each, 5 min on ice between pulses). RNA was purified using the PureLink RNA minikit with the Turbo DNA-free kit (Invitrogen). cDNA was created from 4 µg of total RNA with the SuperScript III first-strand synthesis system (Invitrogen) by using random primers. Quantitative PCR was performed with the FastStart Essential DNA Green Master (Roche) using the LightCycler 96 real-time PCR system (Roche).
