# Supplementary material for: Mycobacterial Caseinolytic Protease Gene Regulator ClgR Is a Substrate of Caseinolytic Protease
Source: mSphere. 2017 Mar 15;2(2):e00338-16. doi: 10.1128/mSphere.00338-16 (PMC5352834; doi:10.1128/mSphere.00338-16)
Supplement: TABLE S1 [file sph002172251st1.pdf]

Table S1. RFP promoter reporter plasmids and primers used in this study.

Plasmid name		Backbone plasmid (Digested with)	Inserted Promoter (PCR-amplified DNA fragments digested with NotI and BamHI/BsaI)				Inserted Reporter (PCR product digested with BamH and EcoRI)	
			Gene	Size (bp)	Primer Name	Primer Sequence	Primer Name	Primer Sequence
1	P-acr2-RFP	pMV306 (NotI---EcoRI)	BCG_0289c	213	P-acr2-F(NotI)	gcggccgcGGTCTGAGGGTATGAGGGGCAAA	mCh-F(BamHI)	ccgggatccATGGTGAGCAAGGGCGAGG
					P-acr2-R(BamHI)	ccgggatccGGTTATCTCCTCATGCTTCGTTGT	mCh-R(EcoRI)	ccggaattcCTACTTGTACAGCTCGTCCAT
2	P-clgR-RFP	pMV306 (NotI---EcoRI)	BCG_2761c	250	P-clgR-F(NotI)	gcggccgcCTGAAGACCTTTGTCCAGGC	mCh-F(BamHI)	ccgggatccATGGTGAGCAAGGGCGAGG
					P-clgR-R(BsaI)	cgggtctcggatccCAAAAACTCCTTGTCCACCTC	mCh-R(EcoRI)	ccggaattcCTACTTGTACAGCTCGTCCAT
3	P-clpP1P2-RFP	pMV306 (NotI---EcoRI)	BCG_2481c	246	P-clpP1P2-F(NotI)	gcggccgcCGACAGTGACGGAAACACGATCG	mCh-F(BamHI)	ccgggatccATGGTGAGCAAGGGCGAGG
					P-clpP1P2-R(BamHI)	ccgggatccAGTGGGGCACCTGCTTTCCTCG	mCh-R(EcoRI)	ccggaattcCTACTTGTACAGCTCGTCCAT
