# Supplementary material for: Mycobacterial Caseinolytic Protease Gene Regulator ClgR Is a Substrate of Caseinolytic Protease
Source: mSphere. 2017 Mar 15;2(2):e00338-16. doi: 10.1128/mSphere.00338-16 (PMC5352834; doi:10.1128/mSphere.00338-16)
Supplement: TABLE S2 [file sph002172251st2.pdf]

Table S2. Primers used for qRT-PCR.

Target	Primer	Primer sequences	Amplicon (bp)	Reference
16S	Fwd	ATGACGGCCTTCGGGTTGTAA	160	Datta 2015 (modified for BCG)
	Rev	CGGCTGCTGGCACGTAGTTG		
acr2	Fwd	ACCGCTGGCTACGTGACTTCTT	139	Datta 2015
	Rev	TCAATGCCGGGCAGTTCCAAA		
clgR	Fwd	CCTCGGGTATCTGTCGGAGAT	84	McGillivray 2014
	Rev	AGCTGCAGAGCCGTACAAATC		
clpP1P2	Fwd	CGCCGTGATCAAGAAAGAAATG	126	McGillivray 2014
	Rev	AAACCGTATTCCAGGGCTTC		

Datta P, Ravi J, Guerrini V, Chauhan R, Neiditch MB, Shell SS, Fortune SM, Hancioglu B, Igoshin OA, Gennaro ML. 2015. The Psp system of Mycobacterium tuberculosis integrates envelope stress-sensing and envelope-preserving functions. Mol Microbiol 97:408-422.

McGillivray A, Golden NA, Gautam US, Mehra S, Kaushal D. 2014. The Mycobacterium tuberculosis Rv2745c plays an important role in responding to redox stress. PLoS One 9:e93604.
