# Supplementary material for: Mycobacterial Caseinolytic Protease Gene Regulator ClgR Is a Substrate of Caseinolytic Protease
Source: mSphere. 2017 Mar 15;2(2):e00338-16. doi: 10.1128/mSphere.00338-16 (PMC5352834; doi:10.1128/mSphere.00338-16)
Supplement: TABLE S3 [file sph002172251st3.pdf]

Table S3. RFP protein fusion plasmids and primers used in this study.

Plasmid name	Backbone plasmid (Digested with)	Inserted Reporters/gene fragments (PCR-amplified DNA fragments)			
		Primer Name	Primer Sequence	Primer Name	Primer Sequence
1 RFP-ClgR	pMV262 (BamHI---HindIII)	mCh-F(BamHI)<br>mCh-Link-R(EcoRI)	ccgggatccATGGTGAGCAAGGGCGAGG<br>ccggaattcACCAGAACCACCCTTGTACAGCTCGTCCATACC	clgR-F(EcoRI)<br>clgR-R(HindIII)	ccggaattcATGGCGGCTTTGGTGCGTGA<br>ccgaagcttTTAGGCCACCGCCAGCGAC
2 ClgR-RFP	pMV262 (BamHI---HindIII)	ClgR-F(BamHI)<br>ClgR-Link-R(EcoRI)	ccgggatccATGGCGGCTTTGGTGCGTGA<br>ccggaattcACCAGAACCACCGGCCACCGCCAGCGACAC	mCh-F(EcoRI)<br>mCh-R(HindIII)	ccggaattcATGGTGAGCAAGGGCGAGG<br>ccgaagcttCTACTTGTACAGCTCGTCCAT
3 eGFP-ClgR	pMV262 (BamHI---HindIII)	mCh-F(BamHI)<br>eGFP-Link-R(EcoRI)	ccgggatccATGGTGAGCAAGGGCGAGG<br>ccggaattcACCAGAACCACCCTTGTACAGCTCGTCCATGC	clgR-F(EcoRI)<br>clgR-R(HindIII)	ccggaattcATGGCGGCTTTGGTGCGTGA<br>ccgaagcttTTAGGCCACCGCCAGCGAC
4 CgR-eGFP	pMV262 (BamHI---HindIII)	clgR-F(BamHI)<br>clgR-Link-R(EcoRI)	ccgggatccATGGCGGCTTTGGTGCGTGA<br>ccggaattcACCAGAACCACCGGCCACCGCCAGCGACAC	eGFP-F(EcoRI)<br>eGFP-R(HindIII)	ccggaattcATGGTGAGCAAGGGCGAGGAGC<br>ccgaagcttCTACTTGTACAGCTCGTCCATGC
5 RFP-ClgR(CΔ29)	pMV262 (BamHI---HindIII)	mCh-F(BamHI)<br>mCh-Link-R(EcoRI)	ccgggatccATGGTGAGCAAGGGCGAGG<br>ccggaattcACCAGAACCACCCTTGTACAGCTCGTCCATACC	clgR-F(EcoRI)<br>clgR(CΔ29)-R(HindIII)	ccggaattcATGGCGGCTTTGGTGCGTGA<br>ccgaagcttTTAGCGGGCAAGGCGCTCTTGA
6 RFP	pMV262 (BamHI---EcoRI)	mCh-F(BamHI)<br>mCh-R(EcoRI)	ccgggatccATGGTGAGCAAGGGCGAGG<br>ccggaattcCTACTTGTACAGCTCGTCCAT		
7 RFP-SsrA	pMV262 (BamHI---EcoRI)	mCh-F(BamHI)<br>ssrA-R(EcoRI)	ccgggatccATGGTGAGCAAGGGCGAGG<br>ccggaattcCTACGCGGCCAGTGCGTA		
8 RFP-ClgR(C9)	pMV262 (BamHI---EcoRI)	mCh-F(BamHI)<br>mCh-clgR(c9)-R(EcoRI)	ccgggatccATGGTGAGCAAGGGCGAGG<br>ccggaattcTTAGGCCACCGCCAGCGACACCACCGGCGCCTTGTACAGC<br>TCGTCCATACC		
9 RFP-ClgR(C5)	pMV262 (BamHI---EcoRI)	mCh-F(BamHI)<br>mCh-clgR(c5)-R(EcoRI)	ccgggatccATGGTGAGCAAGGGCGAGG<br>ccggaattcTTAGGCCACCGCCAGCGACTTGTACAGCTCGTCCATACC		
10 ClgR-RFP-SsrA	CgR-RFP (EcoRI---HindIII)	mCh-F(EcoRI)<br>ssrA-R(HindIII)	ccggaattcATGGTGAGCAAGGGCGAGG<br>ccgaagcttCTACGCGGCCAGTGCGTA		
11 ClgR-RFP-ClgR(C9)	ClgR-RFP (EcoRI---HindIII)	mCh-F(EcoRI)<br>clgR(c9)-R(HindIII)	ccggaattcATGGTGAGCAAGGGCGAGG<br>ccgaagcttTTAGGCCACCGCCAGCGACA		
12 ClgR	pMV262 (BamHI---HindIII)	clgR-F(BamHI)<br>clgR-R(HindIII)	ccgggatccATGGCGGCTTTGGTGCGTGA<br>ccgaagcttTTAGGCCACCGCCAGCGAC		
13 ClgR(CΔ29)	pMV262 (BamHI---HindIII)	clgR-F(BamHI)<br>clgR(CΔ29)-R(HindIII)	ccgggatccATGGCGGCTTTGGTGCGTGA<br>ccgaagcttTTAGCGGGCAAGGCGCTCTTGA		
14 ClgR(CΔ9)	pMV262 (BamHI---HindIII)	clgR-F(BamHI)<br>clgR(CΔ9)-R(HindIII)	ccgggatccATGGCGGCTTTGGTGCGTGA<br>ccgaagcttTTAAATGACGACCTTGGTGCTG		
